# Surgical leadership in changing times: the American College of Surgeons perspective

**DOI:** 10.1515/iss-2019-0007

**Published:** 2019-06-12

**Authors:** Barbara Lee Bass

**Affiliations:** Department of Surgery, John F. Jr. and Carolyn Bookout Presidential Distinguished Chair, Professor of Surgery, Weill Cornell Medical College, Houston Methodist Institute for Academic Medicine, Executive Director, MITIE: Houston Methodist Institute for Technology, Innovation and Education, Houston Methodist Hospital, 6550 Fannin Street, Suite 1661A, Houston, TX 77030, USA

**Keywords:** global surgery, healthcare delivery systems, leadership, professionalism, simulation-based education, surgical education, surgical quality and value

## Abstract

Surgeons around the globe are challenged by the rapid evolution of the environment in which they practice their profession. Changes in surgical technologies, the complexity of surgical patient care, and the regulatory and financial environment of surgical care delivery demand that surgeons be supported in their work with access to superb educational offerings and engagement to foster satisfaction and efficacy in their professional activities. The American College of Surgeons (ACS), the largest international surgical professional organization, is committed to supporting surgeons as leaders in the healthcare system to build programs to create the optimal environment for delivery of quality surgical care to our patients. A selected portfolio of the programs of the ACS is presented.

## Introduction

Unquestionably, the practice of surgery and the environment of care in which surgical care is delivered were in constant evolution and transformation over the last century. Indeed, most would contend that the pace of change and impact of this evolution has accelerated over the last two decades – and many might argue that each year is ever more disruptive. The changes in the surgical environment in the US are far too many to fully articulate: fundamental changes in surgical technologies; the complexity of the surgical conditions we treat, which now commonly require sophisticated interdisciplinary healthcare teams to optimize surgical results; the environment of surgical practice and delivery with increasingly corporate structures and external regulatory oversight. All these factors fundamentally altered the once autonomous and authoritative position of the surgeon caring for a surgical patient.

Coupling these changes to the rapidly advancing body of science that supports our understanding of surgical disease and the escalating challenges to ensure that we, as surgeons, are providing the best value care to our patients can, at times, craft a daunting challenge to the individual surgeon. Further, the new members of the surgical profession are a more diverse group in terms of gender, ethnicity, race, and perhaps most importantly, generational professional expectations than those who raised them in the discipline, differences, which at times create further disruption to the generation of surgeons who precede them.

However, these changes, from the perspective of the ACS, are actually opportunities to continue to evolve and to exercise leadership, and to create platforms and programs to support surgeons as a most vital element of the modern health care system. As stated on the seal of the American College of Surgeons, *“To serve all with skill and fidelity*” and as reiterated in modern framing in our current mission statement, the *American College of Surgeons is dedicated to improving the care of the surgical patient and to safeguarding standards of care in an optimal and ethical practice environment*, the fundamental purpose of our organization has not changed over the now 114-year history of the ACS.

Central to delivering on this mission is the identity of surgeons as leaders, leaders in their practices, leaders in their hospital systems, leaders in education, and leaders in developing quality programs to serve the surgical patient. Inherently, surgeons are placed in an empowered position in their roles of performing surgery for patients, simply by virtue of their acknowledged accomplishments in education and training. While once this empowerment was held with authoritative leadership, the modern form of surgeon leadership empowerment now is a responsibility to serve as a collaborative leader of a high-performance team. The ACS developed many programs to support surgeons in this empowered leadership role to ensure that they are well prepared over their many decades long surgical careers to be able to provide not only high-quality patient care but also, for those who are so placed, to train the next generation of surgeons, to create educational platforms to support lifelong learning for surgeons in practice, to advocate for optimal resources and environments in which surgeons care for their patients, and to advance and contribute to healthcare systems to ensure surgical excellence. Delivering on these goals to optimize the surgeon’s ability to provide quality care to their patients is the core purpose of the ACS.

## Fellowship, quality, value, and safety

Since its founding in 1913 under the guidance of Drs. Franklin Martin and Ernest Codman – the founder of the end results concept, and the Mayo brothers, among others, the ACS has committed to fostering excellence not only by supporting the environment of care but also by articulating the qualities of a skilled and responsible surgeon [1]. Fellowship in the ACS is awarded to those who are highly qualified professionals as evidenced by education, training, examination and board certification, ongoing practice experience, and the professional qualities of commitment to patient primacy, integrity, and service. The review process is rigorous and includes a local assessment of the surgeon’s practice by members of the Membership Committee. Further, deviations from professional standards are reviewed by the Central Judiciary Committee of the Board of Regents, with punitive and dismissal actions taken against Fellows who fail to adhere to the quality and professional standards set by the organization.

**Figure 1:**
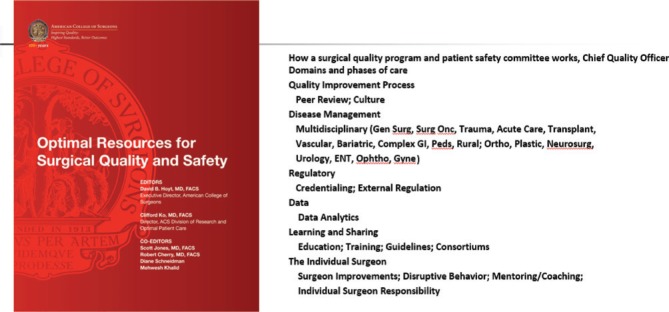
*The Optimal Resources for Surgical Quality and Safety* provides standards for developing measurable parameters of surgical quality and defines infrastructure for quality performance for surgery in a healthcare organization.

Not only does the College set standards for individuals as Fellows, throughout its history the ACS was instrumental in building programs and setting quality standards for the practice of surgery and surgical systems. The first notable effort established criteria for the standards necessary to deliver safe surgical care in the hospital setting – a program now manifest as the Joint Commission, a national and international review organization that certifies hospitals and hospital systems for structure and processes concordant with delivery of quality healthcare. The most recent contribution is a consensus standard setting and accreditation program, *Optimal Resources for Surgical Quality and Safety* developed under the guidance of the ACS Executive Director David Hoyt, MD, FACS, and Director of the Division of Research and Optimal Patient Care Clifford Ko, MD, FACS, to define elements and processes essential to creating a surgical care delivery unit [2] (Figure 1).

The fundamental strategy for these quality programs is to create consensus-driven and evidence-based standards for surgical care; standards related to surgeon qualifications, technical capacity of the environment, processes, organizational structures, and processes of care, and other factors. Once conceived as a set of standards, implementation strategies to build the infrastructure and processes are set, and a quality initiative is born. A department or surgical unit then builds the essential elements and enters the program. Quality clinical data, to allow risk adjustment, comparative measurement, and improvement or decline in performance are essential to the quality and success of all College quality initiatives.

**Figure 2:**
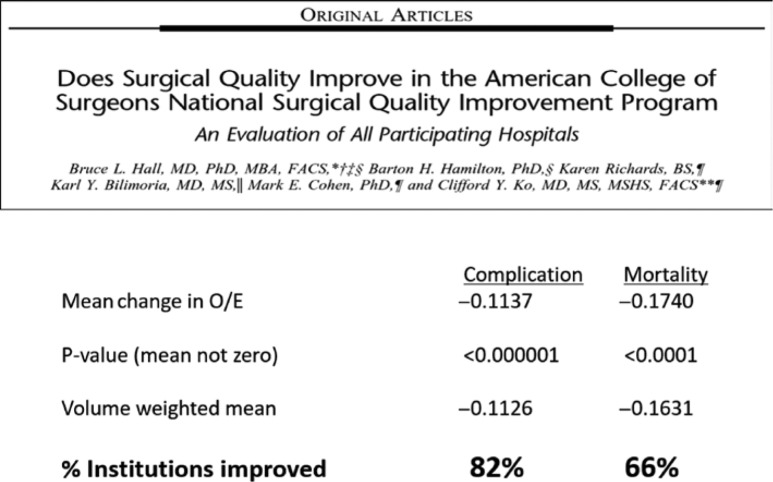
Eighty-two percent of institutions participating in the ACS NSQIP saw highly significant improvements in post-operative complications, and 66% saw a decline in postoperative mortality over the duration of participation.

**Figure 3:**
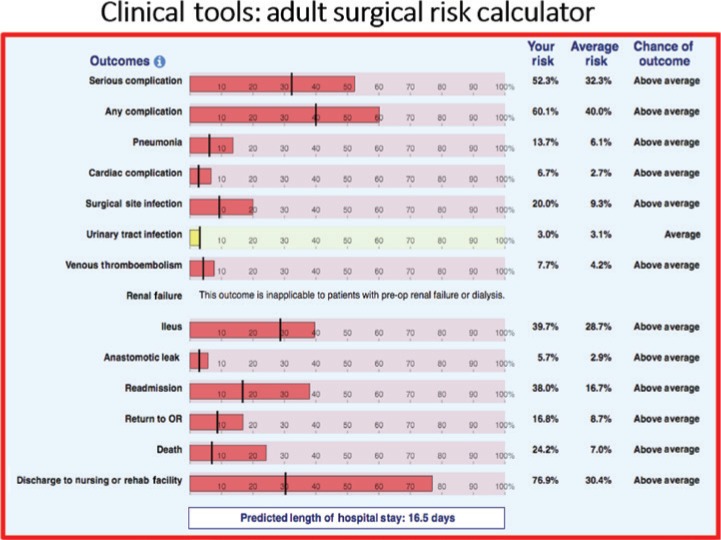
The online NSQIP adult surgical risk calculator allows surgeons to measure estimated post-operative morbidity and mortality risks for individual patients being considered for surgery based on preoperative comorbidity predictive models.

**Figure 4:**
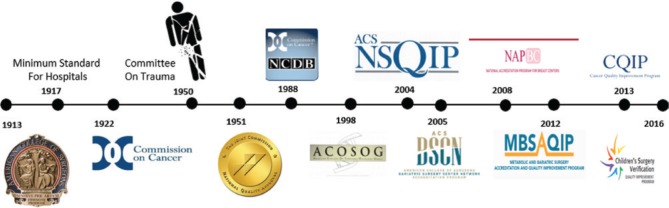
The ACS has been a leader in the development and implementation of data-based surgical quality initiatives over the last century. Landmark programs include the Commission on Cancer, the trauma center verification programs of the Committee on Trauma, the ACS NSQIP, among many others.

Examples of these quality programs and standard setting initiatives include among many others, the products of the ACS Committee on Trauma in developing the Trauma Verification System to create verified Trauma Centers, buoyed by its hallmark educational program, the Advanced Trauma Life Support Course; the Commission on Cancer, which accredits cancer programs for interdisciplinary care, quality of environment and processes, data capture and research initiatives, to optimize the care of patients with cancer; and more recently the development of the ACS National Surgical Quality Improvement Program (NSQIP), a precise clinical data registry system, which allows measurement of risk-adjusted surgical outcomes (Figure 2). Founded in the US Veteran Affairs Healthcare System with the visionary leadership of Shukri Khuri, MD, FACS, the NSQIP is now the world’s largest risk-adjusted surgical outcome measurement tool [3] (Figure 3). The program also developed an online risk-adjustment tool to allow individual surgeons to calculate patient-specific outcomes for planned surgical interventions [4] (Figure 4). In the United States, well over 4000 healthcare organizations and many other international surgical programs, now embrace one or more of these quality programs as a core element of their surgical quality efforts. No other surgical organization in our nation has taken such a leadership position nor committed the organizational resources to build such infrastructure. Furthermore, and in somewhat astonishing fashion, the vast majority of the work of these quality systems is performed by surgeon volunteers, surgeons who are committing their personal time to craft the formation of these programs and to deliver these products to the greater surgical community. A timeline of the quality programs that were developed and are currently led by the ACS is shown in Figure 4.

## Surgeons as leaders

A common thread in the quality initiatives of the ACS and support of surgeons in their careers in all domains is the role of surgeons as leaders. Unlike the authoritative leader of decades past, the new surgical leader requires the skills to engage a diverse group of team members to achieve goals. In this new model of leadership, articulated in every quality program of the ACS, the concept of engaging a team to achieve high-performance is embraced. The role of a leader is to provide the cohesive vision for a team to willingly work together to achieve the mission [5].

In surgery, high-performance interdisciplinary teams are essential to efficient high-quality and safe patient care in the complex environments of the operating room, the trauma bay, the critical care unit, or the multidisciplinary clinic caring for patients with complex disorders of cancer, gastrointestinal disorders, and cardiovascular disease, among many others. Successful leaders in these environments demonstrate emotional intelligence and commitment to clear communication, inclusivity for diverse perspectives and talents, and inclusivity in regard to personal characteristics such as gender, race, ethnicity, and religious differences. While implicit bias does still, at times, confound full inclusion, explicit bias in the surgical professional environment is widely condemned as a dysfunctional and harmful element in an interdisciplinary high-performance team.

## Training surgeons as leaders

Not only has the ACS been at the forefront of developing programs to optimize the care of surgical patients, the College also intentionally developed programs to optimize the personal development of surgeons as leaders.

Examples of leadership training opportunities include an annual symposium on leadership hosted by the ACS in Washington DC. Attended annually by 600–700 surgeons from around the US and the globe, this conference provides didactic and programmatic engagement for surgeons to inspire and prepare them as leaders in their clinical environments, administrative roles, and educators. Crafted by Patricia Turner, MD, FACS, the Director of the Division of Member Services of the College and the young leaders in training of the ACS, the conference addresses principles of leadership, best practices for leadership, and motivational instruction allowing surgeons the opportunity to identify local, regional, and national opportunities to function as leaders in the discipline and in their communities.

For over a decade, the ACS sponsored an exemplary leadership course for mid and senior career surgeons who aspire to major leadership roles in their institutions, academic programs, or other forms of service in the healthcare environment of surgery. This 4-day highly interactive course enabled many surgeons to acquire practical skills to become effective leaders. The program includes instruction on optimization of communication, knowledge of different forms of leadership and appropriateness of such use, identification of times of crisis and optimization of leadership tools during those periods, and the core values of servant leadership and emotional intelligence. Taught by an accomplished group of leaders within the ACS representing academic and healthcare organizations, this program was instrumental in developing a cohesive group of leaders who share the same language and principles to allow them to effectively engage as leaders.

## Education: the core mission

The Division of Education of the ACS, led by Ajit Sachdeva, MD, FACS, developed a remarkable robust set of materials and programs to support surgeons throughout their careers. The ACS has been an effective leading voice in fostering its membership to engage in lifelong learning and has created abundant continuing professional development tools to foster success in that essential domain. The annual Clinical Congress of the ACS attracts roughly 9000 surgeons from the US and globally per year for 4 days of intense educational offerings. The *Journal of the American College of Surgeons* is provided as a benefit of membership to all surgeons, Fellows, and resident associate society members. The Surgical Education Self-Assessment Program (SESAP) has been a core element of self-education for surgeons for the last 30 years. This interactive case-based study program, comprised of 600 questions and a wide spectrum of content, provides exceptional opportunities for surgeons to maintain their surgical knowledge and to self-assess [6].

**Figure 5:**
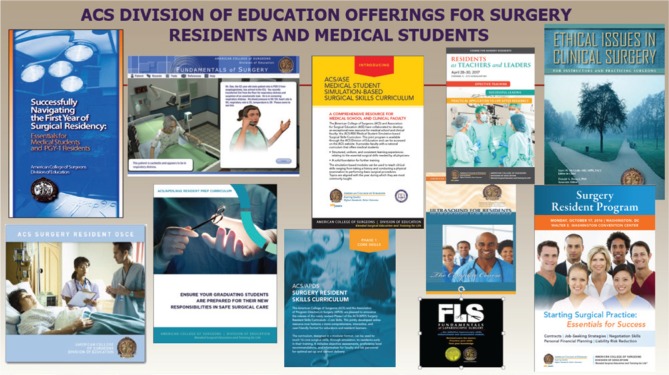
The ACS develops surgical curricula for medical students and surgical residents, including a comprehensive curriculum for simulation-based skills training.

**Figure 6:**
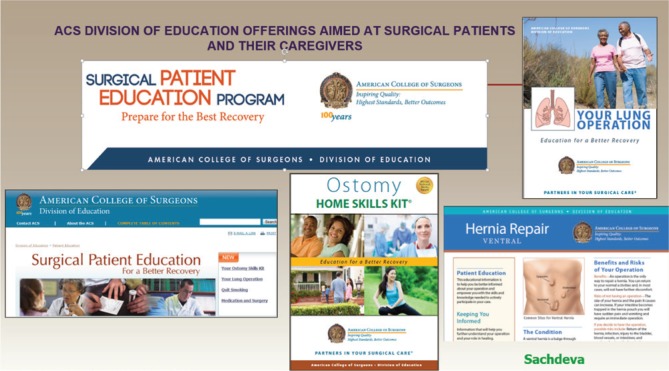
Patients benefit from educational offerings from the ACS including a skills training kit for patients with ostomy.

**Figure 7:**
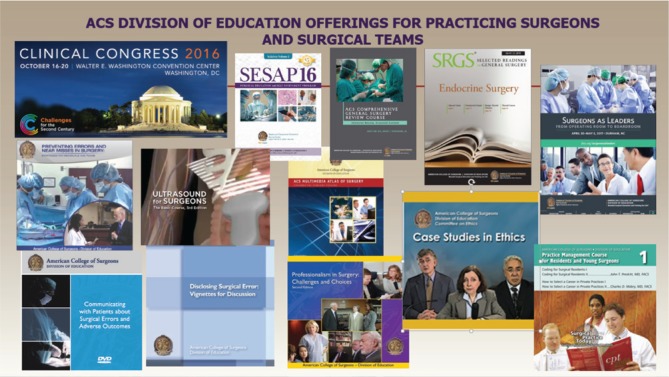
Educational offerings for practicing surgeons and surgical teams produced by The Division of Education under the direction of Ajit Sachdeva, MD, FACS. The Clinical Congress, SESAP, and cross cutting education in surgical ethics, professionalism, and leadership offer outstanding learning opportunities.

With the increasing specialization and expansion of discipline-specific surgical knowledge and expertise, much specialty specific education is now delivered by specialty societies and in specialty journals. Therefore, in addition to its abundant portfolio of educational offerings in the disciplines comprising general surgery, the special role of the ACS as the largest of the worlds’ surgical organizations with well over 80,000 members in the US and globally, has evolved to serve as the leading provider of crosscutting educational content that bridges all surgical disciplines – professionalism, ethics and surgical healthcare policy, communications, palliative care, and medical student surgical education, among many others. The sampling of the abundant portfolio is shown in Figures 5–7.

### Simulation-based education

The ACS was a pioneering leader in embracing simulation-based training for skill acquisition and procedural adoption. The Accredited Educational Institutes of the ACS now hosts 76 centers around the nation and the globe that developed educational centers to leverage simulation-based training in both technical and nontechnical skills training. The centers transformed surgical education starting at the medical student level, through residency, and, in particular, informed and improved the adoption of novel procedures and technologies by surgeons in practice who must continually safely acquire new skills, outside the safe boundaries of experiential learning that is available during residency training. Many of these centers served as vital retooling centers, a new component in the surgical educational landscape, necessitated by the rapid advances in novel technologies and procedures in surgery.

Contributing to the education and development of the surgical workforce of the future was a leading concern for the ACS over the last 20 years. The products are many, including a case-based, web-based interactive curriculum to prepare entering surgical residents for their first year of residency training, development of a fundamental and core surgical skills curriculum for use in simulation training, ATLS, and a number of other didactic and simulation-based courses to optimize educational learning and assessment [6].

## Creating the next generation of surgeon scientists and surgeon educators

The College has long invested in the future of young surgeon scientists with a substantial portfolio of training programs, courses, and scholarships. Courses to train young academic surgeons in health services and outcome science research, grantsmanship, and clinical trial methodology, surgeons as educators at both the faculty and resident level, and mini-fellowship in healthcare policy leadership are hosted annually by the ACS academic Fellows of many disciplines. Further, supported by generous gifts from donors, the College annually awards over $1,500,000 in research scholarships and fellowships to young academic surgeons launching their independent careers as principal investigators; the overwhelming number of these awardees have gone on to lifelong leadership in academic surgery making important contributions to surgical science and education.

The ACS also served as the leading convener in the US of organizations responsible for surgical training. The surgical Residency Review Committees of the Accreditation Council of Graduate Medical Education, the American Board of Medical Specialties surgery boards, program directors for surgical residency training programs, and other groups are all regularly convened at the College as the leading umbrella organization to address evolving issues in surgical training and education. Most recently, these discussions focused on resident autonomy, preparation for independent practice, and work-hour rules for surgeons in training.

## Advocacy initiatives of the American College of Surgeons

As the umbrella organization for all disciplines of surgery, the ACS has served as a primary and invaluable source for keeping its membership prepared for changes in the healthcare delivery system, from developments in healthcare finance and reimbursement to the regulatory environment. Given its size and scale and active presence in the Washington, DC legislative and regulatory environment, the ACS has substantial impact with policy makers to guide regulations and legislation to support the delivery of quality surgical care. The advocacy efforts of the ACS focused on the transition from simply high-quality care to high-value care – care that is both cost and process efficient and of high quality. The College not only advocates for high-value surgical care for the patient’s sake but also embraces its role in preparing the surgical workforce to be optimal performers in the delivery of high-value surgical care. This is a challenging deliverable at times, as the forces that regulate the practice of surgery were viewed with increasing frustration and distrust by many practicing surgeons. The College’s leadership approach in this field is to advocate for initiatives that optimize the ability of a surgeon to provide high-quality and high-value care that will ensure access for all patients to surgical care. Coupled to access and value-driven initiatives are College advocacy efforts to minimize burdensome aspects of the regulatory environment, such as excessive demands of the electronic health record, increasingly restrictive payment models, and increasing corporatization of the healthcare system, which, at times, diminishes the sense of autonomy and effectiveness of surgeons as physicians for their patients.

The ACS, as a leadership organization, must, and consistently does, provide a positive looking strategy of crafting proposals both to benefit the patients and the surgeons in the long run. Certainly, ensuring a surgical workforce that is well, valued, and satisfied in their professional work is an important element of creating a high-value system and the ACS’s advocacy efforts in regard to physician payment, quality metrics, and steps to unburden surgeons from unnecessary regulatory requirements are all important measures to ensure that a surgical workforce is available as needed to meet the needs of our communities.

## Diversity: creating an inclusive professional environment

The face of the American surgical workforce is changing rapidly. The value of having a workforce that reflects the diversity of the community served was well documented to be of benefit to the people of that community. Cultural competency improves the ability of the healthcare workforce, including surgeons, to meaningfully and empathically provide care to the patients they serve.

In the US, for the first time in 2018, women comprised slightly more than 50% of the entering medical student class. Similarly, over the last two decades, women have risen from approximately 10% of the residents in training in general surgery to almost 40% in the last academic year. While other disciplines in surgery were slower to incorporate women into the training pathways, plastic and reconstructive surgery, and colorectal surgery, and urology now also host residency classes with approximately 25%–30% women. Cardiothoracic surgery, orthopedics, and neurosurgery are also beginning to reveal greater numbers of women trainees among their ranks. Underrepresented minorities and surgery are increasingly entering the surgical discipline including Hispanic, Asian American, and African-American trainees [7].

The ACS took many intentional steps to create inclusive environments for this new cohort of diverse surgeons. The ACS now has a committee on diversity and a committee on women in surgery. Intentional inclusion of women and underrepresented minorities in committees within the ACS including the program committee, the scholarship committee, education and policy committees fostered a sense of engagement and inclusion for these newer members of the surgical community. The College adopted statements that take leadership stances on concerns that may limit career opportunities in surgery for women and underrepresented minorities in the profession, including statements on compensation equity, parental leave, and bullying and harassment. Educational programs focused on the role of implicit bias in fostering continued gaps in opportunities and compensation for women and other underrepresented minorities. While many other organizations are now taking similar steps, the ACS, with the leadership of Olga Jonassen, MD, FACS, James Carrico, MD, FACS, Claude Organ, MD, FACS, and others, intentionally embraced and expanded the importance of inclusivity to improve the community of surgeons. This programmatic approach led to advancement of numerous underrepresented minorities and women to leadership positions within the ACS serving as officers of the College, the Board of Regents, Board of Governors, and committee chairs. The visibility of these diverse leaders served as an inspiration for the next generation of surgeons who aspire to successful careers in surgery despite their current minority status [8].

The ACS also embraced active participation and engagement of the future leaders in American surgery by actively recruiting and empowering the resident members and young surgeons in the College. Separate governance structures and committees allow these young surgeons to craft programming pertinent to their interests, to bring their new ideas and concepts forward to the ACS’ governing bodies, and to provide dynamic outreach to medical students and the young surgeon community [6].

Similarly, as the global community of surgery coalesces, the ACS fostered connections and collaborations with surgeons around the globe and developed opportunities for Fellows and young members to serve in low- and middle-income countries. The creation of the program known as Operation Giving Back, currently led by Girma Tefera, MD, FACS, fostered pathways for Fellows to serve as surgeons and collaborators in low- and middle-income countries and to support surgeons to respond to areas of crisis in the setting of US and global surgical needs. Similar to efforts of other European nations, the ACS partnered with the College of Surgeons of Eastern, Central and Southern Africa (COSECSA), including a new partnership with Hawassa University in Ethiopia, a training hospital of the COSECSA network. This collaboration of 13 US academic medical centers under the umbrella of the ACS will provide ongoing support of surgery residency training, surgical quality and research education initiatives, and assist as requested to implement an expanded global surgical training experience for US general surgery residents with the goal of supporting the development of sustainable surgical workforce in this region of the world [9].

## Summary

The modern surgical landscape in the US is rapidly evolving. The greatest challenge is to ensure that high-quality, high-value surgical care is available to all patients in the communities we serve. To that end, a skilled surgical workforce, committed to professional excellence, coupled to high-performance environments of healthcare delivery and surgical care are required. Surgeons with leadership skills, armed with the modern tools of servant leadership, emotional intelligence, and enhanced team performance, are essential.

To deliver on this goal, the Fellows and leadership of the ACS has a 100-year history of leading the way in American surgery to address evolving health care surgical needs, to craft necessary surgical education products, and to foster development of a diverse and effective surgical workforce. These tasks are ever-changing and require surgeons to adapt and to take on new forms of leadership. The many programs, supported by the abundant wisdom and voluntary contributions of the Fellows of the College and the organization’s high-performing executive team, are well prepared to take on these challenges with energy and optimism.

## Supplementary Material

The article (iss-2019-0007) offers reviewer assessments as supplementary material.
