# Supplementary material for: Surgical leadership in changing times: the American College of Surgeons perspective
Source: Innov Surg Sci. 2019 Jun 12;4(2):75–83. doi: 10.1515/iss-2019-0007 (PMC6754053; doi:10.1515/iss-2019-0007)
Supplement: Supplementary file 1 [file iss-4-20190007-s001.pdf]

Original Article

Barbara Lee Bass*

Surgical leadership in changing times: the American College of Surgeons perspective

https://doi.org/10.1515/iss-2019-0007
Received April 17, 2019; accepted April 24, 2019

***Corresponding author: Barbara Lee Bass,** MD, FACS, Chair, Department of Surgery, John F. Jr. and Carolyn Bookout Presidential Distinguished Chair, Professor of Surgery, Weill Cornell Medical College, Houston Methodist Institute for Academic Medicine, Executive Director, MITIE: Houston Methodist Institute for Technology, Innovation and Education, Houston Methodist Hospital, 6550 Fannin Street, Suite 1661A, Houston, TX 77030, USA, E-mail: bbass@houstonmethodist.org. https://orcid.org/0000-0002-9222-9800

Reviewers' Comments to Original Submission

Reviewer 1: anonymous

Apr 21, 2019

**Reviewer Recommendation Term:**	Accept
**Overall Reviewer Manuscript Rating:**	100
Is the subject area appropriate for you?	5 - High/Yes
Does the title clearly reflect the paper's content?	5 - High/Yes
Does the abstract clearly reflect the paper's content?	5 - High/Yes
Do the keywords clearly reflect the paper's content?	5 - High/Yes
Does the introduction present the problem clearly?	5 - High/Yes
Are the results/conclusions justified?	5 - High/Yes
How comprehensive and up-to-date is the subject matter presented?	5 - High/Yes
How adequate is the data presentation?	N/A
Are units and terminology used correctly?	N/A
Is the number of cases adequate?	N/A
Are the experimental methods/clinical studies adequate?	N/A
Is the length appropriate in relation to the content?	5 - High/Yes
Does the reader get new insights from the article?	5 - High/Yes
Please rate the practical significance.	5 - High/Yes
Please rate the accuracy of methods.	N/A
Please rate the statistical evaluation and quality control.	N/A
Please rate the appropriateness of the figures and tables.	4
Please rate the appropriateness of the references.	4
Please evaluate the writing style and use of language.	5 - High/Yes
Please judge the overall scientific quality of the manuscript.	5 - High/Yes
Are you willing to review the revision of this manuscript?	Yes

Comments to Author:
Excellent paper - nothing more to be said!

Open Access. © 2019 Bass B.L., published by De Gruyter. This work is licensed under the Creative Commons Attribution 4.0 Public License.

Reviewer 2: anonymous

Apr 23, 2019

**Reviewer Recommendation Term:**	Accept
**Overall Reviewer Manuscript Rating:**	85
Is the subject area appropriate for you?	5 - High/Yes
Does the title clearly reflect the paper's content?	5 - High/Yes
Does the abstract clearly reflect the paper's content?	5 - High/Yes
Do the keywords clearly reflect the paper's content?	5 - High/Yes
Does the introduction present the problem clearly?	5 - High/Yes
Are the results/conclusions justified?	5 - High/Yes
How comprehensive and up-to-date is the subject matter presented?	5 - High/Yes
How adequate is the data presentation?	5 - High/Yes
Are units and terminology used correctly?	5 - High/Yes
Is the number of cases adequate?	N/A
Are the experimental methods/clinical studies adequate?	N/A
Is the length appropriate in relation to the content?	5 - High/Yes
Does the reader get new insights from the article?	5 - High/Yes
Please rate the practical significance.	5 - High/Yes
Please rate the accuracy of methods.	N/A
Please rate the statistical evaluation and quality control.	5 - High/Yes
Please rate the appropriateness of the figures and tables.	5 - High/Yes
Please rate the appropriateness of the references.	5 - High/Yes
Please evaluate the writing style and use of language.	5 - High/Yes
Please judge the overall scientific quality of the manuscript.	5 - High/Yes
Are you willing to review the revision of this manuscript?	No: Not necessary

Comments to Author:

This well-written paper gives an excellent comprehensive overview of a special aspect of surgical leadership which is relevant for almost all countries. In the authors opinion the greatest challenge is to ensure that high-quality, high-value surgical care is available to all patients in the communities we serve. Therefore surgical leadership does not only comprise as leadership skills of the individual surgeon. It also means, that surgical scientific societies must take the lead in the development and implementation of data based surgical quality initiatives. The ACS NSQIP, the online NSQIP adult surgical risk calculator, the surgical curricula for medical students and surgical residents, and the educational offerings for practicing surgeons and surgical teams are programs of the American College of surgeons without equal. After the introduction of ACS NSQIP a verifiable quality improvement could be demonstrated by reducing post-operative complications combined with a decline in postoperative mortality over the duration of participation.
